# Association between inflammatory biomarkers and adiposity in obese patients with heart failure and metabolic syndrome

**DOI:** 10.3892/etm.2014.1673

**Published:** 2014-04-08

**Authors:** MARJAN MOTIE, LORRAINE S. EVANGELISTA, TAMARA HORWICH, DAWN LOMBARDO, FRANK ZALDIVAR, MICHELE HAMILTON, GREGG C. FONAROW

**Affiliations:** 1 Program of Nursing Science, University of California Irvine, Irvine, CA 92697, CA, USA; 2 Division of Cardiology, David Geffen School of Medicine, University of California at Los Angeles, Los Angeles 90095, CA, USA; 3 Division of Cardiology, Department of Medicine, University of California Irvine Medical Center, Orange 92868, CA, USA; 4 Institute for Clinical and Translational Science, University of California Irvine Medical Center, Orange 92868, CA, USA; 5 Cedars-Sinai Heart Institute, Los Angeles 90048, CA, USA

**Keywords:** heart failure, biomarkers, C-reactive protein, leptin, obesity

## Abstract

Obesity, type 2 diabetes mellitus (DM) and metabolic syndrome (MS) are common in patients with heart failure (HF). Studies investigating the association between known biomarkers and adiposity in patient populations are limited. The aim of the present study was to investigate the association between C-reactive protein (CRP) and leptin with adiposity in a sub-group of overweight/obese patients with HF, DM and/or MS. A total of 36 patients (mean age, 56.72±9.78 years; ranging between 27 and 76 years of age; 80.6% male; 52.8% Caucasian) were enrolled and their height, weight, waist circumference and body composition (e.g. percentage body fat and lean mass), as well as the levels of CRP and leptin, were assessed. The results demonstrated that there was a significant association between CRP and leptin, CRP and body mass index (BMI) and gender and percentage body fat (P<0.05, for all associations). Analysis of leptin and CRP levels revealed that patients in the highest BMI quartile (BMI, 40.3–61.2) had higher CRP levels (4.83 μg/ml vs. 3.03 μg/ml; P=0.033) and higher leptin levels (44.97 ng/ml vs. 24.64 ng/ml; P=0.042) compared with patients in the lower BMI quartile (BMI, 28.6–32.4). In conclusion, among obese patients with HF, DM and/or MS, an association between CRP and leptin was identified, providing further evidence that metabolic and inflammatory mechanisms are involved in these diseases. Future investigation to assess the potential impact of inflammation and adiposity, and the role of dietary interventions and weight loss on clinical outcomes in this population of chronically ill patients is warranted.

## Introduction

Heart failure (HF), obesity and type 2 diabetes mellitus (DM) are disease conditions associated with the progression of cardiovascular disease, functional disability and a diminished quality of life as reported in our recent study (1). Previous studies have also indicated that the coexistence of all three conditions leads to the combined synergistic interaction of pathophysiological responses associated with each condition resulting in a higher morbidity and mortality rate (2–7). Numerous contributing factors have been hypothesized to be involved in obesity-linked metabolic and inflammatory alterations associated with cardiovascular disease. Two of these factors that have been implicated in each of these disease states are C-reactive protein (CRP) and leptin.

CRP is a marker of systemic inflammation and the levels of CRP markedly increase as part of the acute-phase response. CRP is synthesized by the liver and regulated by cytokines, particularly interleukin (IL)-6 (7). Elevated levels of CRP have been found to be associated with the development of coronary heart disease and metabolic syndrome (MS) (8–11). Current evidence suggests that inflammation and biomarkers, including high-sensitivity CRP, may be as important as cholesterol in determining the development of atherosclerosis and heart disease. By contrast, whilst obesity is one of the strongest determinants of CRP levels (12), the exact mechanism linking obesity and inflammation remains to be elucidated.

Leptin, an adipocyte-derived hormone is involved in appetite regulation, insulin homeostasis and obesity through the effects of the hypothalamus (13,14). Leptin levels may be used to predict the development of MS independent of obesity (15). Furthermore, leptin has also been implicated in contributing to an increased risk of cardiovascular disease with or without coexisting atherosclerosis (16–18). It has been proposed that leptin may induce the expression of CRP in vascular endothelial cells leading to possible deleterious effects (19). This theory is supported by studies reporting a significant correlation between levels of leptin and CRP in a variety of patient populations (20–25). A number of studies have also investigated the involvement of CRP in ‘leptin resistance’ (the coexistence of elevated leptin levels in obese individuals despite the role of leptin in decreasing appetite and reducing food intake) (26–28). There are, however, clear gaps in the understanding of the correlation between leptin and CRP in terms of whether leptin affects CRP expression directly or through independent mechanisms, and to what extent the interactions directly affect the onset and course of disease.

The aim of the present study was to determine the circulating levels of CRP and leptin and to examine their correlation with each other as well as with body mass index (BMI) and adiposity in overweight/obese patients with HF, DM and/or MS. Investigation of these biomarkers may provide insight into the association between inflammatory and metabolic mechanisms involved in disease onset and pathophysiology in this patient population.

## Patients and methods

### Study population

A total of 36 subjects (>18 years old) were recruited and provided informed consent to participate in a randomized controlled clinical trial for overweight/obese patients with HF, DM and/or MS. The present study was approved by the Institutional Human Subjects Review Committee. Inclusion criteria included a BMI >27, New York Heart Association (NYHA) functional class II or III HF (either with preserved left ventricular ejection fraction [HFpEF] or reduced ejection fraction [HFrEF]) and a history of DM (e.g. impaired fasting glucose of 100–125 mg/dl), or meeting three or more of the criteria for MS. Participants were excluded if they were pregnant, had a serum creatinine level >1.5 mg/dl or had a history of clinically significant illness, including acute myocardial infarction or sustained ventricular arrhythmia three months prior to the start of the experiment. In addition, patients were also excluded if they were currently suffering from liver, respiratory and/or gastrointestinal disease and malignancy, or a history of gout. Participants were interviewed by nurses to provide information regarding sociodemographic and clinical variables (including, age, gender, history of illness and medication).

### Weight, BMI and waist circumference

Height, weight, waist circumference and vital signs were measured by the research staff as previously described (1). Body composition was measured by having participants complete a whole-body scan with dual-energy X-ray absorptiometry (Hologic 4500A, version 12.3; Hologic, Inc., Waltham, MA, USA), which provided assessments of fat, lean and bone mineral mass by measuring the differential absorption of X-rays and utilizing the computer algorithms provided by the manufacturer. Percentage body fat was calculated as the ratio of fat mass relative to overall body weight.

### Laboratory measurements

Following an overnight fast, venous blood samples were obtained and the levels of total cholesterol, high-density lipoprotein, low-density lipoprotein, triglycerides, fasting blood glucose and insulin were measured. High sensitivity CRP and leptin levels were measured using Quantikine Elisa Assay kits purchased from R&D Systems (Minneapolis, MN, USA). CRP was measured with lower and upper detection limits of 0.78 ng/ml and 50 ng/ml, respectively, using an assay sensitivity of 0.022 ng/ml. The assay for leptin measured between 16 and 1,000 pg/ml, with a sensitivity of 7.8 pg/ml.

### Statistical analysis

Patients were classified into four BMI (kg/m^2^) categories based on quartiles of all BMI data collected in the present study: i) BMI 28.6–32.4, ii) BMI 32.5–35.8, iii) BMI 35.9–40.2 and iv) BMI 40.3–61.2. Data were analyzed using SPSS statistical software version 19.0 for Windows (SPSS, Inc., Chicago, IL, USA). Analysis of variance was used to compare sociodemographic and clinical variables of the entire sample size (n=36), as well as each of the BMI quartiles. Comparison of CRP and leptin levels within gender or BMI groups was performed using the independent samples t-test. The association between serum CRP and leptin levels and age, gender, waist circumference, total percentage fat and total cholesterol were evaluated using Pearson’s product moment correlation coefficient and Spearman’s Rho, depending on the level of measurement. Multivariate analysis was performed to test the independent association between CRP and leptin using leptin as the dependent variable and CRP, age, gender, BMI, waist circumference and statin as covariates.

## Results

### Participant characteristics

Table I shows the sociodemographic and clinical characteristics of the study sample. Comparative data for the four BMI quartiles are also illustrated in the table. Participants ranged between 27 and 77 years old (average age, 56.72±9.8 years), and were predominantly male (80.6%). On average, participants were moderately obese (weight, 113.78±25.57 kg; BMI 37.6±7.2 kg/m^2^) and had a mean total cholesterol of 158.5±39.6 and a total body fat percentage of 36.9±7.0%. With the exception of the expected weight-associated variables (weight, BMI and waist circumference), the participants in the four BMI quartiles only differed significantly in the mean age (due to the inclusion of a 27 year old in the highest quartile).

### Association between CRP and leptin and other cardiovascular risk factors

Univariate analyses for variables of interest are illustrated in Table II. BMI was associated with age (P=0.001) and CRP (P=0.043). The association between BMI and age is an outcome of significantly lower mean age in the highest BMI quartile (Table I) compared with all the other BMI quartiles. A strong correlation between CRP and leptin was also observed (P=0.001). In the multivariate analysis, leptin was independently associated with CRP, following adjustment for age, gender, BMI, waist circumference and whether or not the patient was using lipid lowering statin drugs (F=1.05; P=0.002). A statistically significant correlation between gender and total body fat (P<0.001) and CRP (P=0.02) was identified. Gender differences between CRP, leptin and percentage body fat are illustrated in Table III. Females had significantly higher levels of CRP and percentage body fat compared with males.

The different BMI quartiles were then compared in order to further evaluate the association between obesity and levels of CRP and leptin. While the association between leptin and CRP was independent of obesity level, there was a statistically significant increase in the concentration of these markers as the level of obesity increased (Table IV). In the most obese patients (BMI, 40.3–61.2), CRP and leptin were significantly higher compared with the least obese patients (BMI, 28.6–32.4).

## Discussion

To the best of our knowledge, the present study is the first to examine the association between CRP, leptin and adiposity and other cardiovascular risk factors in overweight/obese patients with HF, DM and/or MS. The moderately strong association between CRP and adiposity observed in the present study is in accordance with a previous study that linked adiposity with a chronic state of inflammation, which may be involved in the development of multiple chronic illnesses, including cardiovascular disease and MS (29). Furthermore, inflammation in the presence of obesity is hypothesized to arise primarily in adipose tissue due to alterations in metabolic homeostasis leading to increased cytokine production and the activation of inflammatory signaling pathways in the body (30). Therefore, the importance of weight loss, accompanied by reductions in percentage body fat, requires further investigation in this population to evaluate the usefulness of CRP as a prognostic marker of inflammation and disease state in a subgroup of overweight/obese patients with HF, DM and/or MS.

In the present study, a statistically significant correlation between CRP and leptin was identified, independent of BMI in the present study population of overweight/obese patients with HF, and this has also been confirmed in a number of other patient populations and healthy adults (20–25). The marked increase revealed in the concentration of these markers as the level of adiposity increased provides further support for an association between these inflammatory biomarkers and obesity. Whilst studies investigating the association between CRP and adiposity are widespread in various populations (8–11,29), the association between leptin and obesity appears to be more complex. Leptin was previously considered to only be involved in energy balance and satiety. However, it has now been demonstrated that leptin receptors are located throughout the body, including the heart, suggesting that it is also involved in the regulation of other processes (31). Recently, it has been suggested that leptin may act as a cardiac hypertrophic factor linked to obesity and that concurrent elevated leptin levels are associated with cardiovascular risk, particularly in patients with HF (32).

The mechanisms linking CRP with leptin in different disease models is not well understood. Since CRP synthesis is considered to be regulated by IL-6 (7), and since leptin secretion from human adipose tissue also stimulates the production of various cytokines (including IL-6), it has been hypothesized that leptin may regulate CRP production (20,21). In obesity, CRP has also been postulated to be involved in the development of leptin resistance (26–28). The clinical significance of the association between CRP and leptin, and the possible additive effects of these measures of inflammation/immunomodulation as they are associated with an increased cardiovascular risk, warrants further investigation.

The results from the present study demonstrated that levels of CRP were significantly higher in females compared with males; however, differences in leptin levels between males and females were not observed. It was also found that females had a greater percentage body fat compared with males. A study of ~2,750 individuals, aged between 30 and 65 years (>50% female) found that CRP levels were almost twice as high in females compared with males (3.3 vs. 1.8 mg/l; P<0.05) (33). In a recent study Khera *et al* (34) investigated gender differences in the association between CRP and body fat. Previous studies have also revealed that the quantity and distribution of body fat affects CRP to a greater extent in females compared with males. Therefore, the results from the present study are in accordance with the hypothesis that adiposity, associated with subclinical inflammation, may be of particular importance in females. The question remains, however, as to the value of CRP in assessing the risk of cardiovascular disease in males compared with females. While CRP levels are higher in females compared with males, males develop heart disease more frequently and have a higher positive correlation with CRP compared with females (35,36). Gender differences were evaluated in the National Health and Nutrition Examination Survey, and it was demonstrated that, while males with elevated levels of CRP (>3.0 mg/dl) had increased cardiovascular mortality and all-cause mortality hazards (defined by hazard ratios or HR), this difference was not observed in females, leading to the conclusion that there is a requirement to tailor recommendations on diagnostic and prognostic use of CRP based on gender (37). Future investigation is required to illustrate the differences in gender and CRP as it is associated with obesity in females and males to further clarify the importance of this inflammatory marker in cardiovascular and associated risk factors. The findings of the present study regarding the correlation of CRP with leptin in overweight/obese patients with HF, DM and/or MS, and the lack of gender differences in the levels of leptin may indicate the potential effectiveness of concurrent use of these biomarkers in prognosis and designing of weight-loss interventions to affect clinical outcomes in this patient population.

The present study was limited by the number of participants enrolled and the fewer numbers of females compared with males. Furthermore, there is always a limitation in analyzing multiple factors in biological systems (e.g. leptin, CRP and obesity) due to the adjustments that have to be made for common disease pathways. Finally, analysis of the current diet and physical activity of each participant may potentially alter the risk estimates used in the present study. In future, a larger sample size is required and the effect of weight loss regimens need to be evaluated to further reveal the link between inflammation, adiposity and adipokines, and their association with risk factors and outcome in overweight/obese patients with HF, DM and/or MS.

## Figures and Tables

**Table I:** Sociodemographic and clinical characteristics (n=36).

		BMI quartile				
			
Characteristic	All participants (n=36)	(i) 28.6–32.4 (n=9)	(ii) 32.5–35.8 (n=10)	(iii) 35.9–40.2 (n=8)	(iv) 40.3–61.2 (n=9)	P-value
Age, (years)	56.72±9.78	60.78±6.61	57.90±5.11	60.00±11.08	48.44±11.40	0.021
Male, n (%)	29 (80.6%)	8 (88.9%)	8 (80.0%)	7 (87.5%)	5 (55.6%)	0.298
Diastolic blood pressure (mmHg)	73.14±13.23	74.56±13.36	66.80±6.39	70.25±15.31	81.33±14.13	0.095
Systolic blood pressure (mmHg)	120.72±23.84	120.44±14.98	105.50±14.20	126.00±34.28	133.22±22.64	0.066
Patient taking statins (%)	27 (75.0%)	7 (77.8%)	8 (80.0 %)	8 (100 %)	4 (44.4%)	0.062
Weight (kg)	113.78±25.57	96.87±7.41	101.99±10.88	108.10±10.90	145.61±26.85	<0.001
BMI (kg/m^2^)	37.64±7.24	30.90±1.21	34.43±1.13	37.40±1.69	48.15±5.95	<0.001
Waist circumference (cm)	47.41±4.84	43.61±2.89	45.92±2.26	47.44±3.80	53.18±3.77	<0.001
Total % fat (DEXA)	36.88±6.98	30.52±4.42	35.17±6.53	38.62±3.54	44.42±4.72	<0.001
Fasting glucose (mg/dl)	130.09±51.29	110.67±22.45	156.60±50.66	133.78±73.04	130.34±50.55	0.299
Total cholesterol	158.50±39.64	151.24±35.98	158.80±41.45	137.02±29.86	184.55±39.64	0.081
LDL	91.97±38.25	79.54±32.92	90.30±34.31	86.48±26.42	110.89±49.07	0.326
HDL	40.68±11.06	41.20±13.08	38.90±5.74	39.98±11.48	42.78±13.07	0.889
Triglycerides	154.33±78.71	160.33±90.65	157.90±92.47	124.63±43.56	170.78±79.79	0.676
Leptin (ng/ml)	40.34±24.67	24.64±12.47	46.96±29.06	44.54±25.75	44.97±24.55	0.178
CRP (μg/ml)	4.06±2.12	3.03±1.40	4.16±2.28	4.20±2.72	4.83±1.85	0.347

The data are presented as the mean ± standard deviation. BMI, body mass index; DEXA, dual-energy X-ray absorptiometry; LDL, low density lipoprotein; HDL, high density lipoprotein; CRP, C-reactive protein.

**Table II:** Correlational matrix of the key variables of interest.

Characteristic	BMI	Age	Gender	Waist circumference	Percent body fat	Cholesterol	Leptin	CRP
BMI								
Age	−0.521^a^							
Gender	−0.216	0.262						
Waist circumference	0.667^a^	−0.199	−0.001					
Percentage body fat	0.692^a^	−0.289	−0.610^*^	0.520^a^				
Cholesterol	0.300	−0.194	−0.034	0.363^b^	0.118			
Leptin	0.246	0.074	−0.113	0.257	0.173	0.264		
CRP	0.340^b^	−0.265	−0.382^b^	0.205	0.426^b^	0.195	0.525^a^	

a P<0.01 (2-tailed) and

b P<0.05 (2-tailed).

BMI, body mass index; CRP, C-reactive protein.

**Table III:** Gender differences between CRP, leptin and percentage body fat.

Characteristic	All participants (n=36)	Males (n=28)	Females (n=8)	P-value
CRP (μg/ml)	4.06±2.12	3.63±2.13	5.55±1.29	0.022
Leptin (ng/ml)	40.34±24.67	38.87±24.97	45.49±24.47	0.511
Total % fat	36.88±6.98	34.59±5.87	44.58±4.53	<0.001

CRP, C-reactive protein.

**Table IV:** Comparison of CRP and leptin levels in the lowest and highest BMI quartiles.

		BMI quartile		
			
Characteristic	All participants (n=36)	(i) 28.6–32.4 (n=9)	(iv) 40.3–61.2 (n=9)	P-value
CRP (μg/ml)	4.06±2.12	3.03±1.40	4.83±1.85	0.033
Leptin (ng/ml)	40.34±24.67	24.64±12.47	44.97±24.55	0.042

BMI, body mass index; CRP, C-reactive protein.
